# Effect of different visual presentations on the comprehension of prognostic information: a systematic review

**DOI:** 10.1186/s12911-021-01612-9

**Published:** 2021-08-25

**Authors:** Eman Abukmail, Mina Bakhit, Chris Del Mar, Tammy Hoffmann

**Affiliations:** Faculty of Health Sciences and Medicine, Institute for Evidence-Based Healthcare, Bond University, 14 University Dr, Robina, QLD 4229 Australia

**Keywords:** Prognosis, Natural history, Health communication, Decision support techniques

## Abstract

**Background:**

Understanding prognostic information can help patients know what may happen to their health over time and make informed decisions. However, communicating prognostic information well can be challenging.

**Purpose:**

To conduct a systematic review to identify and synthesize research that has evaluated visual presentations that communicate quantitative prognostic information to patients or the public.

**Data sources:**

MEDLINE, EMBASE, CINAHL, **PsycINFO**, ERIC and the Cochrane Central Register of Controlled Trials (CENTRAL) (from inception to December 2020), and forward and backward citation search.

**Study selection:**

Two authors independently screened search results and assessed eligibility. To be eligible, studies required a quantitative design and comparison of at least one visual presentation with another presentation of quantitative prognostic information. The primary outcome was comprehension of the presented information. Secondary outcomes were preferences for or satisfaction with the presentations viewed, and behavioral intentions.

**Data extraction:**

Two authors independently assessed risk of bias and extracted data.

**Data synthesis:**

Eleven studies (all randomized trials) were identified. We grouped studies according to the presentation type evaluated. Bar graph versus pictograph (3 studies): no difference in comprehension between the groups. Survival vs mortality curves (2 studies): no difference in one study; higher comprehension in survival curve group in another study. Tabular format versus pictograph (4 studies): 2 studies reported similar comprehension between groups; 2 found higher comprehension in pictograph groups. Tabular versus free text (3 studies): 2 studies found no difference between groups; 1 found higher comprehension in a tabular group.

**Limitations:**

Heterogeneity in the visual presentations and outcome measures, precluding meta-analysis.

**Conclusions:**

No visual presentation appears to be consistently superior to communicate quantitative prognostic information.

**Supplementary Information:**

The online version contains supplementary material available at 10.1186/s12911-021-01612-9.

## Introduction

Shared decision making is a bidirectional communication process in which clinicians and patients collaborate on making a health decision and discuss the available options (based on the best available evidence), the benefits and harms of each option, and the patient’s values, preferences, and circumstances [1, 2]. As part of making decisions about the prevention or management of a health condition, patients need to know about more than just treatment options; they also need to know about the prognosis of the condition, with and without treatment.

Communication of prognostic information is essential as it helps patients to know what may happen to their health over time, to make appropriate preparations, and to make informed decisions about whether to intervene and if so, how. If prognostic information is not communicated adequately, patients may have inaccurate expectations about the likely course of their illness [3–5]. Poor communication of prognostic information can also make patients anxious, confused, and damage the relationship between clinician and patient [6, 7].

Prognosis communication can be complex and challenging for clinicians and patients. Clinicians sometimes try to avoid or delay this kind of communication, while patients often wait for their clinicians to initiate the process [8]. As with the communication of treatment information, a contributor to the challenge of communicating prognostic information well is the difficulty that clinicians and patients can have understanding relevant quantitative information [9–11].

To facilitate discussion about prognosis, clinicians may use visual means (such as a graph) to present the quantitative information. Although there is a large body of synthesized evidence on how to communicate the quantitative benefits and harms of treatments, we are unaware of any synthesis about the various visual presentations that can be used to communicate quantitative prognostic information.

## Methods

The protocol of this systematic review is registered at (CRD42020192564) and can be found in the Open Science Framework osf.io/ze26g.

### Objective

This systematic review aimed to identify and synthesize research that has evaluated visual presentations that communicate quantitative prognostic information to patients or the public.

### Information sources

We searched for studies in six databases: MEDLINE, EMBASE, CINAHL, ERIC, PsycINFO, and the Cochrane Central Register of Controlled Trials (CENTRAL), each from date of inception till December 2020. We used a tailored search strategy for each database (see Additional file 1). Forward and backward citation analysis of the included studies was performed using Web of Science.

### Eligibility criteria

#### Study types and participants

We included only studies with a quantitative design that looked at the prognosis of any health condition (real condition, hypothetical scenario, or fictitious condition). The only participation restriction was the exclusion of health professionals or health professional students. Studies of mixed populations (e.g. health professionals and patients) were eligible if data were reported separately for the eligible group. There was no limitation on study setting or publication language.

#### Interventions

Studies were eligible if they compared a visual presentation (e.g. a graph, words and numbers displayed in tabular format) with at least one another type of presentation (e.g. another type of graph, or free text) to display quantitative prognostic information and included a specified time frame (e.g. over the next 5 years) or time point (e.g. at 1 year). Prognostic information was defined as information about the likelihood of any future outcome in patients with a given health condition including those who received no treatment (natural history) or those who did.

#### Outcomes

Our primary outcome was comprehension of the presented information that was assessed using questions that required a quantitative answer (e.g. likelihood or duration of the outcome). For this reason, only some of the questions asked were eligible. Studies/data that assessed comprehension with questions requiring qualitative responses were ineligible. Secondary outcomes included: preferences for any of the presentations evaluated; satisfaction with the presentation; and behavioral intentions relevant to the information presented (e.g. intention to be screened).

### Study selection

Two authors (EA and MB) independently screened the titles and abstracts, and then the full text of potentially included studies. Discrepancies were resolved through consultation with the other two senior authors (CDM and TH).

### Data extraction and risk of bias assessment

Data were extracted into a custom-designed spreadsheet. The studies’ characteristics (e.g., study settings, sampling methods, study design) and participants’ characteristics (e.g., age, sex, educational level, health literacy level, numeracy level, and the health condition studied). Intervention details (including type of presentation (e.g., bar graph), the presented information, who delivered the information, how, where and when the information was delivered), outcome details (including the eligible outcomes, how they were measured and at what timepoints) and result details (including number of responses analysed, follow up rate, results of eligible studies) are tabulated in the Additional file 1 and show the data extracted. Two authors (EA and MB) independently extracted relevant data and assessed risk of bias of included studies using the Cochrane Risk of Bias tool for randomized trials—version 2 (RoB2) [12]. Any discrepancies either in data extraction or risk of bias assessment were resolved by consulting the other two senior authors (CDM and TH).

### Analysis

Due to heterogeneity of the primary outcome measures (comprehension) and visual presentations used, we were unable to conduct a meta-analysis and therefore report the results narratively. The percentage of participants who answered each eligible question correctly are reported separately for each question and we calculated the average percentage correct across the eligible questions in each study. In studies that did not report the percentage correct for an individual question, we extracted the overall percentage correct for all comprehension questions as reported in the studies.

### Modifications from the protocol

After reviewing the articles generated from our search, we added explicit exclusion criteria that were not detailed in our protocol. Studies that compared alternative statistical formats (e.g. relative risk reduction vs. absolute risk reduction) and studies that compared the framing (i.e. positive or negative) of health information were excluded as they have been previously synthesized [13, 14]. Also excluded were studies that only compared methods of wording free text for conveying prognostic information. The eligibility of the primary outcome measure was also clarified to include questions that required participants to perform a quantitative calculation and then select the answer from categorical or dichotomous response options. The search strategy (see Additional file 1) was slightly modified by adding more MeSH terms (e.g., Comprehension, Knowledge, Data Display, Communication, Perception, “Decision Making, Shared”) at the request of reviewers during the peer-review process. This resulted in no additional eligible studies.

## Results

### Study selection

**Fig. 1:**
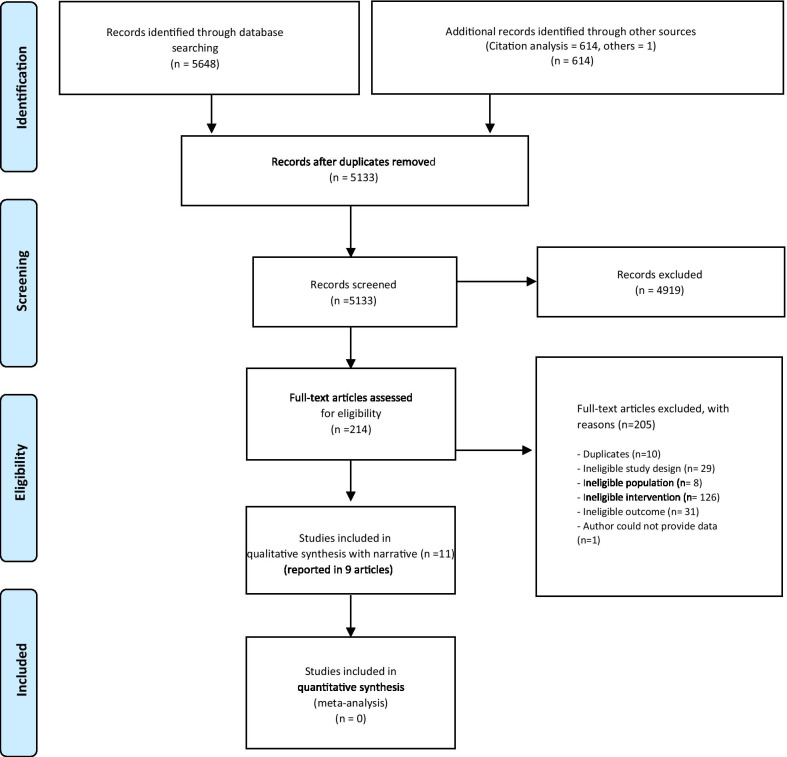
PRISMA flow chart of systematic search and selection

Our search identified 5648 articles across the databases and 614 articles identified through forward and backward citation analysis of the included studies (6262 in total), 5133 remained after duplicates were removed. After the full-text screening, we identified 9 articles: in 2 of these, 2 separate studies were reported, resulting in 11 eligible studies [15–23] (Fig. 1).

### Characteristics of included studies

All 11 included studies were randomized trials. Seven studies were conducted in the United States, 3 in Germany, and 1 in the United Kingdom. The total number of participants was 9737 (mean 885, range 120 to 2305). All participants were adults; 4 studies included only men, 3 studies included only women, 3 included both, and 1 did not report this. Eight studies were conducted online and 3 face-to-face. Health information in the interventions related to the prognosis of cancer (9 studies), middle ear infection (1 study), and multiple sclerosis (1 study). Details of the included studies are presented in the Table of characteristics (see Additional file 1).

### Risk of bias assessment

**Fig. 2:**
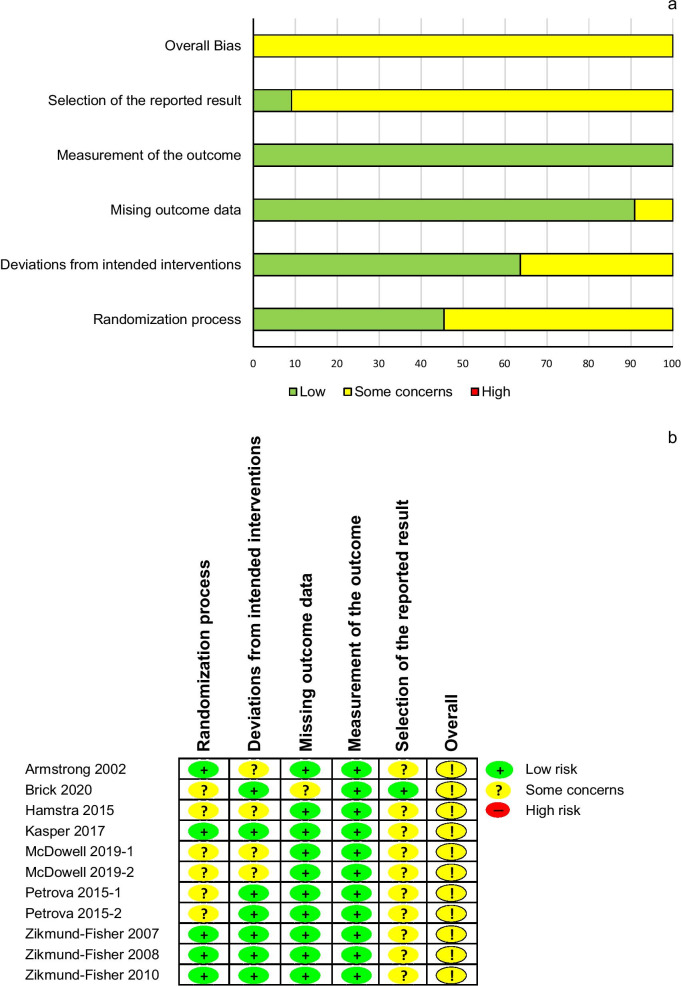
Risk of bias assessment of the included studies. **a**: overall, **b**: individual studies

The overall assessment of the risk of bias of the 11 studies was judged at “some concerns” level. All included articles were judged to have “some concerns” for at least one domain of risk of bias. Ten studies had “some concerns” for the selection of reported results and six had “some concerns” for their randomization process. (Fig. 2a, b).

### Visual presentations evaluated

**Fig. 3:**
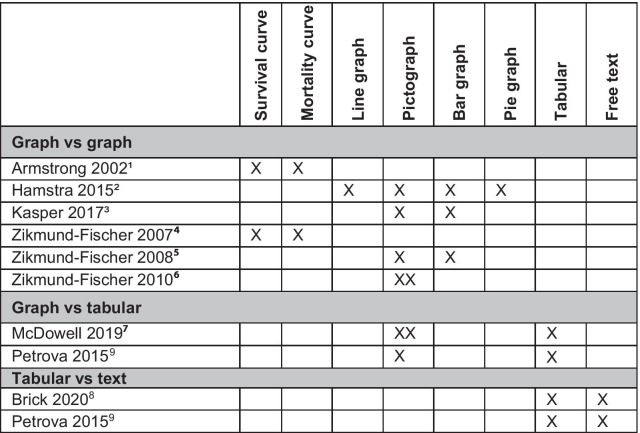
Interventions compared in the included studies. ^1^A third group received both survival and mortality curve. ^2^Eight interventions with variation of each format type were tested (3 pictographs, 2 bar graphs, 1 line graph, 2 pie graphs). ^3^Four interventions: 2 static (bar graph, pictograph) and 2 animated (bar graph, pictograph). ^4^Survival curves were delivered either in 5y or 15y worth data and the same for mortality curves. ^5^Four interventions were tested (4-options pictograph, 4-options bar graph, 2-options pictograph, 2-options bar graph). ^6^Two pictographs; a graph with survival only outcome versus a graph with multiple outcomes. ^7^The three formats were embedded in fact box format, two studies were reported one face to face and one online. ^8^Two conditions were tested in this study; only one contained prognostic information (middle ear infection) and was included. ^9^Two studies were reported (breast cancer screening, female participants) and (prostate cancer screening, male participants). Each study tested 3 interventions (pictograph, tabular, free text). X refers to a study group

Details of the visual presentations evaluated are provided in the Additional file 1 (Table of interventions). Studies were grouped into 4 categories according to the presentation type they used: 6 studies compared a graphic format to another graphic format: 3 studies compared a bar graph to a pictograph/icon array, 2 studies compared survival curves to mortality curves, and 1 study compared two variations of pictographs (the last is covered in “other comparisons”. Four studies compared a graphic format to a tabular format (also known as a ‘fact box’), Three studies compared a tabular format to free text. (Fig. 3).

### Primary outcome: comprehension

#### Graph versus graph (6 studies)

##### Bar graph versus pictograph (i.e. icon array) (3 studies)

**Fig. 4:**
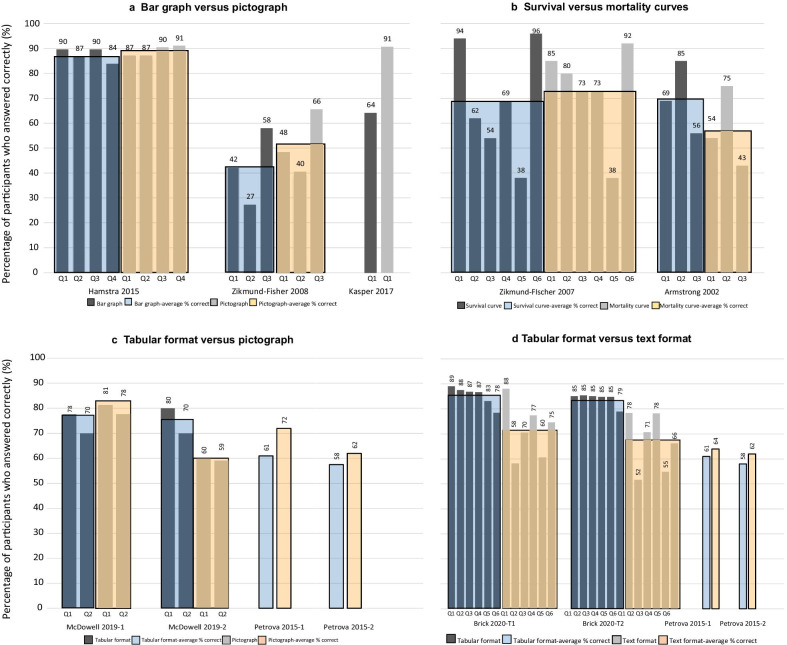
**Comprehension results of visual presentation comparisons** (**a**-**d**). Graphs are different in each study (see Table of interventions in the Additional file 1). Questions are different in each study, for exact wording (see Table of outcomes in the Additional file 1). Kasper 2017 has 1 eligible question. Petrova 2015-1 and 2 reported the overall comprehension per format

Three studies compared bar graphs to pictographs (see Fig. 4a). There was no statistically significant difference in comprehension between these types of graphs [17, 18, 21]. One of the studies enrolled 420 men and randomized them to 1 of 8 graph variations communicating the likelihood of recurrence of prostate cancer over 3-time points (including: 3 pictographs and 2 bar graphs variations). It found that 89% of participants who viewed pictographs (regardless of the variation shown) answered comprehension questions correctly compared to 87% of those who viewed bar graphs (regardless of the variation shown) [17].

In a study on breast cancer prognosis, 1619 participants were randomized to 1 of 4 graph variations (2 pictographs, 2 bar graphs), 51% of participants who viewed pictographs (regardless of the variation) answered comprehension questions correctly compared to 42% who viewed bar graphs (regardless of the variation) [21]. In a study with 682 people with multiple sclerosis, participants were randomized to 1 of 4 graph variations (2 pictographs, 2 bar graphs). The question about prognosis was answered correctly by 91% of those who viewed the pictograph, compared to 64% who viewed the bar graph [18].

##### Survival curves versus mortality curves (2 studies)

Two studies measured comprehension after presenting participants with either survival curves or mortality curves (Fig. 4b). A study on the communication of breast cancer prognosis analyzed responses of 1461 participants and found that there was no difference in comprehension regardless of whether a survival or mortality curve was presented [22]. A study of 451 participants, using a colon cancer scenario, found that those who viewed survival curves scored significantly better than those who viewed mortality curves, with an average correct difference of 13%. A group of participants in the same study who were shown both survival and mortality curves performed slightly better than the group who only saw mortality curves, but the difference was not significant [15].

#### Graph versus tabular format (i.e. fact box) format (4 studies)

Four studies compared pictographs to a tabular format (Fig. 4c). Two studies (reported in the same article, one conducted face-to-face and one online) used three groups to compare a tabular format alone, to a tabular format plus a single pictograph (that showed both benefits and harms), to a tabular format plus two pictographs (one showing benefits and one showing harms). In both studies, the authors reported that the interventions had a similar effect in facilitating comprehension. An average of 74% of participants who viewed the tabular format in the face-to-face study (75% in the online study) compared to 80% who viewed the pictographs (results from both pictograph formats combined) (60% in the online study) correctly answered comprehension questions [20].

Another article reported two studies: a study communicating prostate cancer screening information for male participants and a study communicating breast cancer screening for female participants. The study about prostate cancer found that the use of a pictograph format significantly improved comprehension (*P* = 0.003) compared to a tabular format, with 72% and 61% of participants answered correctly, respectively. Similar results were reported in the breast cancer study, with 62% of those who viewed the pictograph were able to answer the questions correctly compared to 58% who viewed the tabular format [19].

#### Tabular format versus text (3 studies)

Three studies compared a tabular format to a free text format (Fig. 4d). Two studies (reported in the same article) found no significant difference between using a tabular format and free text to communicate prostate cancer prognosis to male participants and prognosis of breast cancer to female participants. In the prostate cancer study, 64% of participants who viewed the text format compared to 61% who viewed the tabular format answered the questions correctly. In the breast cancer study, 61% of those who viewed the text format answered correctly compared to 60% of those who viewed tabular format [19]. A study communicating prognosis of acute middle ear infection with and without antibiotics found that participants who saw a tabular format scored significantly higher on comprehension questions than those who saw a free text format (85% vs 72% correctly answering) [16].

#### Other comparisons

Static formats were better understood than animated formats when displayed online in a German web-based study of 682 people with multiple sclerosis [18]. Displays that contained less information were generally better understood than those with more as found in two studies conducted by the same author [21, 23]. One of these found that pictographs with information about the outcome of two treatment alternatives, compared to those with four, were significantly better understood [21]. In the other study, pictographs that presented data for only one outcome (survival only; the number of women alive after 10 years who had treatment) were significantly better understood than pictographs that presented information about multiple outcomes (survival, mortality due to cancer, mortality due to all causes) [23].

### Secondary outcomes

#### Format preference (1 study)

In the study that randomized participants to 1 of 8 formats (3 pictographs, 2 bar graphs, 1 line graph, 2 pie graphs), participants preferred the bar graph and thought they would understand it better than other formats. The pictograph was rated the lowest on both preference and expected understanding, regardless of the format they were randomized to. Overall, there was no statistically significant difference between graph preference and comprehension in this study [17].

#### Satisfaction (2 studies)

In a study that compared 4 visual presentations (2 bar graph variations, 2 pictograph variations), both the pictograph formats (4-options pictograph and 2-options pictograph) received statistically significantly higher satisfaction scores compared to the 4-options bar graph. However, even though participants were significantly more satisfied with the 2-options bar graph compared to the 4-options bar graph, the satisfaction scores were not as high as for the pictograph formats [21]. In another study, a pictograph that contained only survival data had significantly higher satisfaction scores than a pictograph showing multiple outcomes [23]. Participants of the study involving middle ear infection prognostic information reported being more engaged with the tabular format compared to the free text format [16].

#### Behavioral intentions (4 studies)

Participants in the communication of breast cancer prognosis study who viewed the survival-only pictograph were statistically significant less likely to say that they would have both chemotherapy and hormonal therapy compared to those who viewed the multiple outcome pictograph (*P* = 0.04) [23]. In a 3-arm study that used colon cancer information to compare survival curves, mortality curves, and both curves, participants who viewed survival curves were more likely to choose a preventive colectomy than an annual exam compared to the other two groups [15]. In the study of providing middle ear infection prognostic information, the format did not affect participants’ recommendation to a family member to use antibiotics [16]. Two studies on the communication of prostate cancer prognosis screening outcomes found no association between the type of presentation and the intention to be screened for prostate cancer at any measurement time point in two studies (one conducted online, one conducted face to face) [20].

## Discussion and conclusion

### Discussion

Our main finding is that from the existing studies there does not appear to be a single type of visual presentation that is consistently superior over another for improving the comprehension of quantitative prognostic information for members of the public. In the few studies that examined this, simpler formats (such as one outcome instead of multiple, and fewer intervention options presented at one time) were generally better understood and achieved higher levels of satisfaction. The impact of various types of visual presentations on behavioral intention is inconsistent.

Many primary studies and reviews [9, 10, 13, 14, 24–31], have investigated various methods of communicating treatment benefits and harms. While there are similarities between the communication of treatment benefits and harms and the communication of prognosis information, the extent to which methods identified as superior for communicating treatment quantitative information are also suitable for facilitating the comprehension of prognosis information is unknown. Similar to the findings of a review of methods of communicating quantitative treatment information [9], we found little difference between bar graphs and pictographs in facilitating comprehension and that there is no superior single method for conveying quantitative information.

Research on the comprehension of information from a survival curve with different variations found that comprehension was generally good across each variation [32]. Our review found inconsistent findings of the comparative effect of survival and mortality curves for the comprehension of prognosis information. However, we only identified two studies that had examined this. Details of the chosen curve, such as the complexity of information and the time frame used in the curve, maybe as important as the type of curve [33–37].

The strengths of our systematic review arise from the rigorous method of systematically identifying, screening, and reviewing the relevant literature. Our search was not limited by language; however, studies that do not have an English language title or abstract in the databases might have been missed. Although we searched six databases and conducted citation analysis of the included studies, we may have missed eligible studies. A meta-analysis was precluded due to heterogeneity of the included studies as they used many variations of visual presentations and comprehension was assessed with different measures. Most of the included studies were conducted online, using hypothetical scenarios with participants who did not have the condition being presented. Studies involving participants with the condition of interest may generate different impacts on comprehension, satisfaction, and decisions.

Few primary studies have compared the effectiveness of different visual presentations on the comprehension of quantitative prognostic information and more are needed. Most of the existing studies used cancer scenarios and so future research that explores other conditions would address this research gap, as would head-to-head studies that compare the different presentations. As the superiority of any single visual presentation was not established in this review, visual presentations should be co-designed and piloted with the target population before widespread use.

### Conclusions

From the existing research, there is inconsistency about the superiority of a particular visual presentation to use when discussing quantitative prognostic information with patients. Any of the existing visual presentations that were identified in this review may be suitable to use to aid comprehension.

### Practice implication

Any of the visual presentations identified may be suitable to aid clinicians in discussing prognostic information with patients or their carers. More primary research is needed to identify how patients or the general public understand the prognostic information. Piloting any newly developed tool to communicate prognosis with the target population is highly recommended.

## Supplementary Information

**Additional file 1**: the additional file includes: Search strategy, Table of characteristics, Table of interventions,Table of outcomes, and Table of excluded studies.

## Data Availability

The protocol of this review is registered at PROSPERO (CRD42020192564) and can be found in the Open Science Framework osf.io/ze26g. More information is provided in the Additional file 1. The datasets used and/or analysed during the current study are available from the corresponding author on reasonable request.
