# Supplementary material for: Effect of different visual presentations on the comprehension of prognostic information: a systematic review
Source: BMC Med Inform Decis Mak. 2021 Aug 25;21:249. doi: 10.1186/s12911-021-01612-9 (PMC8390199; doi:10.1186/s12911-021-01612-9)
Supplement: Supplementary file 1 — Additional file 1: the additional file includes: Search strategy, Table of characteristics, Table of interventions,Table of outcomes, and Table of excluded studies. [file 12911_2021_1612_MOESM1_ESM.pdf]

Search strategy*

Ovid MEDLINE

(exp Prognosis/ or Natural history.ti,ab. or Natural course.ti,ab. or Clinical course.ti,ab. or survival.ti,ab. or Watchful waiting.ti,ab. or (Wait adj1 see).ti,ab. or Active monitoring.ti,ab. or Prognosis.ti,ab. or Progress.ti,ab. or Progressing.ti,ab. or Progression.ti,ab. or recurrence.ti,ab. or ((Symptom or Symptoms or Illness or Disease or Condition) adj3 (resolve or resolution or Duration or Length or Time or recurrence or Relapse or Remit or Worsen or Improve or Improves or Improving or stabilize or prevent)).ti,ab.)
and
(exp Communication/ or (Communication or Communicate or Framing or Shared decision making or Perceptions or Perceive or Display or Displays or Displayed or Perceived).ti,ab.)
and
(exp comprehension/ or exp knowledge/ or exp perception/ or (knowledge or understand or Understanding or understandable or comprehend or Comprehends or comprehension or decision or Decisions or describe or Described or Illustrate or Illustrates or Illustrative or Convey or Conveys or Patient preference or Patient education).ti,ab.)
and
(exp Decision Support Techniques/ or exp "Decision making, shared"/ or exp data display/ or (Decision aid or Decision aids or Graph or Graphs or Graphic or Graphics or Graphical or Pictograph or Pictographs or Format or Formats or Curve or Curves or Chart or Charts or Icon array or icon arrays or Histogram or histograms or Numeracy or numerical or Relative risk or Absolute risk or probability information or numerical presentation or Graphical presentation).ti,ab.)

ERIC

(Prognosis OR "Natural history" OR "Natural course" OR "Clinical course" OR survival OR "Watchful waiting" OR (Wait AND see) OR "Active monitoring" OR Prognosis OR Progress OR Progressing OR Progression OR recurrence OR ((Symptom OR Symptoms OR Illness OR Disease OR Condition) AND (resolve OR resolution OR Duration OR Length OR Time OR recurrence OR Relapse OR Remit OR Worsen OR Improve OR Improves OR Improving OR stabilize OR prevent)))
AND
(Communication OR Communication OR Communicate OR Framing OR "Shared decision making" OR Perceptions OR Perceive OR Display OR Displays OR Displayed OR Perceived)
AND
(comprehension OR knowledge OR perception OR knowledge OR understand OR Understanding OR understandable OR comprehend OR Comprehends OR comprehension OR decision OR Decisions OR describe OR Described OR Illustrate OR Illustrates OR Illustrative OR Convey OR Conveys OR "Patient preference" OR "Patient education")
AND
("Decision Support Techniques" OR "Decision making, shared" OR "data display" OR "Decision aid" OR "Decision aids" OR Graph OR Graphs OR Graphic OR Graphics OR Graphical OR Pictograph OR Pictographs OR Format OR Formats OR Curve OR Curves OR Chart OR Charts OR "Icon array" OR "icon arrays" OR Histogram OR histograms OR Numeracy OR numerical OR "Relative risk" OR "Absolute risk" OR "probability information" OR "numerical presentation" OR "Graphical presentation")

*Other databases search strings can be found in our protocol osf.io/ze26g

Table of characteristics

Author, year<br>Registration ID/PMID<br>Country	Objectives of the study	a-Settings<br>b-Sampling method	Study design	Participants<br>a-Number of participants (response rate%)<br>b-Health condition<br>c-Age in years (mean (SD) or median (range))<br>d-Sex (% male)<br>e-Educational level<br>f-Health literacy<br>g-Numeracy
Armstrong 2002<br>(NR, 11833668)<br>USA	- to determine whether framing of information as survival curves or mortality curves affects understanding of the information or the preferred alternative,<br>-whether presenting both survival and mortality curves reduces framing effects.	-Community (individuals awaiting jury duty at the Philadelphia City Courthouse)<br>-individuals are randomly selected for jury duty from voter registration and drivers' license records	RCT, participants were randomised to receive 1 of 3 questionnaires: survival curve only, mortality curve only, both	a-451 (NR)<br>b-hypothetical colon cancer.<br>**mortality group, both curves, survival group**<br>c-Mean age (range), 42.9 (18-79), 42.6(20-76), 41.2 (20-72)<br>d-Female, 70%, 65%,72%<br>e-Mean years of education (range) 13.7 (8-17) 14.1 (9-19) 13.4 (8-17)<br>f- Health literacy NR
Brick 2020<br>(NA, 32269779)<br>UK	test whether table or text-only formats lead to better comprehension of the potential harms and benefits of different options, here in a medical context.	-Online survey panel firm YouGov.<br>-unclear, but UK representative sample	RCT, longitudinal experiment, factorial 2X2, 2 formats, 2 medical conditions: 2formats (tables (fact box), free text), medical conditions: therapeutic (acute middle ear infection), preventative (flu vaccine, **ineligible to our SR**)	a-2305 (NR)<br>b-middle ear infection **(eligible to our SR**), flu **(ineligible to our SR)**<br>-all other demographics were NR, but adjusted to be representative of UK population
Hamstra 2015<br>(NR, 25277673)<br>USA	Assess different graphical formats for imparting knowledge about the longitudinal risks of prostate cancer recurrence with or without 'hormone' or 'androgen deprivation' therapy.	-Academic medical centre<br>- recruited in the waiting areas (family surgical waiting area, cafeteria, etc.) of an academic medical centre located in a moderate-sized US city<br>-NR	RCT, Participants were randomized to 1 of 8 risk communication messages that differed in format: line graph with 1 number, Pie graph with 1 number, bar graph with 1 number, Pie graph with 2 numbers, bar graph with 2 numbers, 1 number, 2 numbers, pictograph with no numbers.	a-420 (NR) men with no history of prostate cancer<br>b- likelihood of prostate cancer returning or spreading<br>c-Median age 52y (36-63)<br>d-100% male<br>e-Education n/%: high school or less 54/13, trade school 17/4, some college 116/29, bachelor's degree or more 218/54<br>f- Health literacy NR<br>g-Numeracy (4% missing), median (IQR) 5.25 (4.5-5.625)<br>High (5-6) 231/55, medium (4 to<5) 130/31, low (1 to<4)44/10.5
Kasper 2017<br>(NR, 27981688)<br>Germany	Evaluate a new space-saving format, CLARIFIG (clarifying risk figures), aiming to facilitate accuracy of comprehension.	-online/ accessed from the starting page of the German MS Self-help Society website (DMSG).<br>-a representative sample of people with MS	RCT (web-based) four arm trial: MFP static, MFP animated, CLARIFIG static, CLARIFIG animated	a-682 (77%) were randomised, 658 completed the study<br>b-Multiple sclerosis (MS)<br>c-Mean age (SD) 40.1 (10.9)<br>**N (%)**<br>d-Female 485 (71%)<br>e-University level education 47 (29.7%)<br>f-Health literacy NR<br>g-Numeracy 2.04 (1.1)
McDowell 2019<br>(NR, 30799691)<br>Germany	study 1<br>examine which of the fact box formats leads to greater comprehension, better short-term knowledge recall, and more informed decision intentions.	-Lab (Max Planck Institute for Human Development participant pool to complete the study in the lab)<br>-Unclear (MPI participant pool)	RCT, Participants were randomly assigned to 1 of 3 fact box formats on prostate cancer screening: a tabular fact box with numbers, a fact box with numbers and icon array, and a fact box with numbers, separate icon arrays, and text to describe each benefit and harm.	a-120 (NR)<br>b-Prostate cancer<br>c-Mean age/SD (range) 51.7/16.5 (30-75)<br>d-100% male<br>e-Education % (years)<br>middle or secondary education 27 (9-10y), high school 23 (12-13), bachelor's or more 45,<br>f-Health literacy NR<br>g-NR

Table of characteristics

Author, year<br>Registration ID/PMID<br>Country	Objectives of the study	a-Settings<br>b-Sampling method	Study design	Participants<br>a-Number of participants (response rate%)<br>b-Health condition<br>c-Age in years (mean (SD) or median (range))<br>d-Sex (% male)<br>e-Educational level<br>f-Health literacy<br>g-Numeracy
McDowell 2019-2	Study 2<br>-whether designing fact boxes to present multiple outcomes with icon arrays would increase their efficacy<br>-examine how fact box formats improved relative to prior knowledge and following a short- or longer-term delay	-online survey company<br>-recruited to participate from a representative pool of German men	RCT, participants were randomly assigned to receive 1 of 3 fact box formats (same as in study 1) or the standard information.	a-244 (347 started survey, 280 randomised)<br>b-Prostate cancer (screening)<br>c-Mean age/SD (range) 53.9/8.7 (40-75)<br>d-100% male<br>e-Education % (years)<br>middle or secondary school 69, high school 27, bachelor's degree or higher 19<br>f-Health literacy NR<br>g-Numeracy score 3,2,1,0 (40.6, 27.5, 17.6,14.3)
Petrova 2015<br>(NR, 26044208)<br>USA<br><br>Experiment 1	Investigate psychological factors that promote the comprehension of benefits and harms associated with common cancer screenings and their influence on shared decision making.	-Web panel Amazon.com Mechanical Turk<br>-diverse computer-literate US residents	RCT, information was provided in 1 of 3 formats: text, a fact box, or a visual aid	a-256 (NR)<br>b-PSA-prostate cancer screening<br>c-mean age/SD (range) 36/13 (18-70)<br>d-100% Male<br>e-Education %<br>completed high school or less 12, at least some college education 27, 2-year college degree 13, 4-year college degree 35, master's degree or higher 13<br>f-Health literacy NR<br>g-Numeracy mean/SD 4.48/ 1.71
Petrova 2015<br>Experiment 2	Same as experiment 1	Same as experiment 1	Same as experiment 1	a-355 (NR)<br>b-mammography based breast cancer screening<br>c-mean age/SD (range) 38/14 (18-85)<br>d-100%females<br>e-NR<br>f-Health literacy NR<br>-3% had been diagnosed with breast cancer<br>g-NR
Zikmund-Fisher 2007<br>(NR, 17137743)<br>USA	Assess if using mortality graphs would reduce people's perception of treatments as less effective when survival graphs show fewer years of data versus more years of data	-online Survey sampling international (SSI)<br>-Stratified sample, participants were drawn from a panel of SSI users who voluntarily agreed to participate in research surveys, one third from each range of ages 21-40,41-59, 60 or more	RCT (factorial 2x2x2), participants were randomized to view either survival or mortality graphs that showed either 5 years of data or 15 years of treatment outcomes data.	a- 1704 randomised; 1461 people (86%) provided useable data.<br>b-a hypothetical condition (Crawford's disease or Crawford's cancer)<br>c-mean (range), 50 (21-89)<br>d-49% males<br>e-bachelor's or higher 37%, high school or less 20%<br>f-Health literacy NR<br>g-Numeracy NR
Zikmund-Fisher 2008<br>(NR, 19012353)<br>USA	If using simpler formats would improve comprehension of the relevant risk statistics compared to presenting the same information in the format currently used in the Adjuvant! tool.	- online Survey sampling international (SSI)<br><br>- participants were women aged 40-74 years who were drawn from a panel of Internet users administered by Survey Sampling International (SSI) and who voluntarily agreed to receive invitations to fill out questionnaires.<br><br>- E-mail invitations were sent to a stratified random sample of panel members	RCT, used internet-administered survey<br>subjects were randomly assigned by computer to 1 of 4 experimental conditions<br>1-a base version that mirrored the Adjuvant! Format<br>2-a graph that used a pictograph format: included 4 pictographs, 1 for each treatment option<br>3-bar graph that showed only 2 options<br>4-pictograph that showed only 2 options	a-1648 women (73%)<br>b-Breast cancer (hypothetically)<br>c-mean (SD)/median 54.5(8.6)/54<br>d-100% female<br>e-Education n (%) ≤high school diploma 404(25), some college 779(48.2), ≥bachelor's college 434(26.8)<br>f-Health literacy NR<br>g-Numeracy Score, 1-6 4.14 [1.13]/4.38<br>-prior breast cancer diagnosis 69 (4.3)

Table of characteristics				
**Author, year<br>Registration ID/PMID<br>Country**	**Objectives of the study**	**a-Settings<br>b-Sampling method**	**Study design**	**Participants<br>a-Number of participants (response rate%)<br>b-Health condition<br>c-Age in years (mean (SD) or median (range))<br>d-Sex (% male)<br>e-Educational level<br>f-Health literacy<br>g-Numeracy**
Zikmund-Fisher 2010<br>(NR, 20375419)<br>USA<br>Primary study	To test whether simpler formats that present only baseline and incremental survival would improve comprehension of the relevant risk statistics and/or affect treatment intentions.	-online<br>- stratified random samples of women ages 40 to 74 y recruited from an Internet research panel (SSI)	RCT, Internet-administered survey<br>Participants were randomized to view either pictographs (icon arrays) that displayed all 4 possible outcomes or pictographs that showed only survival outcomes.	a-838 (75%) analysed out of 857 completed the survey<br>b-Breast cancer (imagine being diagnosed)<br>c-Age y median (range), 54 (40-74) mean/SD 54.7/8.7<br>d-100% female<br>e-Education n (%)<br>High school diploma or less 205 (24.5), Some college 392 (46.8), Bachelor's degree or more 240 (28.7)<br>f-Numeracy score (range, 1–6) 4.22 (1.11)/4.38<br>f-Health literacy NR<br>g-Numeracy NR
Zikmund-Fischer 2010<br>USA<br>Replication study	Same as primary	Same as primary	Same as primary	a-714 (54.6%)<br>b-Breast cancer<br>c-f-demographics (omitted for brevity) mirrored very closely those of the primary study sample.

Table of interventions

**Author, year**	**Interventions**<br>Brief Name<br>type (graph, text) with details	**Outcome in the methods and time point/period**<br>(ex: survival rate over 10years)	**Co-intervention**<br>(Preceded by training for the participants (yes/no)<br>-involved explanation (yes/no), if yes how and by whom)	**Who**<br>delivered the intervention?<br>If provided (their expertise, background, any specific training given)<br>**How**<br>Mode of delivery (face-to-face, Online, Telephone)<br>Individually or in a group	**When and how much**<br>Over what period, no of sessions, duration	**Where**<br>the intervention was delivered
Armstrong 2002	1-survival curves<br>2-mortality curves<br>3-both curves<br>Each intervention contained:<br>1-preexplanation<br>2-a graph: showing two curves of both approaches/treatments<br>3-4 knowledge questions	Number of people surviving/dying out of 100 over years after decision, comparing colectomy and yearly exam vs yearly exam, every 5y for 50y	-Yes, they received a practice graph showing a single curve<br>-Each questionnaire began with a brief explanation of survival and/or mortality curves<br>-For the questionnaire presenting both curves, an explanation was provided about the relationship between the number of people alive (based on the survival curve) and the number of people dead (based on the mortality curve) at any point in time.	-A research assistant<br>-Face to face<br>-individually to a group in a single room	1 session<br>Duration is NR	County Courthouse
Brick 2020	-2X2 (2 messages/conditions, 2 formats) randomised trial<br>Participants randomised to<br>1-fact box summary: brief tabular presentation<br>2-text only summary (control)<br>-2 conditions were tested: acute middle ear infection (eligible here) and flu vaccination (not eligible here)	Benefits and harms of either antibiotics or placebo over a period of 7-14 days<br><br>Pain for 4-7 days after diagnosis<br>Impaired hearing for 6 weeks after diagnosis<br>Perforated eardrum (duration not defined in the Q but in the title of the table)	-No<br>-Prior to seeing the summary, a brief vignette explained the context of the disease, treatment, and efficacy studies.	-NR<br>-Online (via survey company YouGov)<br>-individually	T1 at the start of study, T2 6 weeks later<br>-1 session<br>-about 12 min<br>Average time for completion of the survey in their pilot study was about 7 min	Online: Invitations were emailed
Hamstra 2015	Participants were randomized to 1 of 8 risk communication messages that differed in format, and if the graph included numbers: no numbers (**Pictograph**), 1 number of affected individuals only (**Bar graph 1, Line graph 1, Pie graph 1, Pictograph 1),** Two numbers (both the number affected and the number unaffected (**Bar graph 2**, **Pie graph 2**, **Pictograph 2)**<br>-Participants were presented by a hypothetical scenario then viewed 2 graphs<br>-data about each risk were displayed at 1, 2, and 3 years.	Likelihood of prostate cancer returning or spreading in the next 1, 2, 3 years	No<br>-Explanation provided as text	-NR<br>-Face to face<br>-Individually	One session<br>Duration NR	waiting areas (family surgical waiting area, cafeteria, etc.) of an academic medical centre
Kasper 2017	four arm randomized controlled trial<br>-CLARIFIG presents a sequence of three didactic steps condensed into one proportional bar graph with additional stick figure icons indicating the particular condition of the group represented by each segment of the bar graph<br>-the CLARIFIG graphs show patients experiencing benefit, patients who worsen despite treatment and patients who do not benefit because the intended result would have occurred naturally<br>-The 4 formats: **Gp1-MFP static, Gp2-MFP animated, Gp3-CLARIFIG static, Gp4-CLARIFIG animated**	Progression of MS with and without interferons over 2 years (benefits)	-No<br>-Briefing the participants for the coming information example, a short presentation (three charts) was then provided	-NR<br>-online<br>-individually	One session<br>**Time to complete the survey**<br>**Seconds/SD**<br>MFP static 162.49/99.7<br>animated 285.74/172.11<br>CLARIFIG static 155.89/75.89<br>dynamic 188.45/62.16	Online
McDowell 2019 Study 1	Participants were randomly assigned to receive<br>1 of 3 formats<br>Fact box: tabular format<br>Fact box: single icon array<br>Fact box: separate icon arrays	Benefits and harms of prostate cancer screening, presented as number of men died from prostate cancer out of 1000 with/without detection for 11 years	-No<br>-Yes, the same introductory text was provided for each fact box.<br>All materials were presented in German.	-NR<br>-unclear (face to face, in lab)<br>-individually	One session/duration NR<br>Participants completed comprehension after randomisation to formats while formats were presented<br>And short-term knowledge recall, after short delay (average = 7min)	Max Planck Institute, lab
McDowell 2019 Study 2	Participants were randomly assigned to receive 1 of 3 fact box formats (see study 1) or the standard information	Same as study 1	-No<br>-same as study 1	-NR<br>-Online (contacted by email)<br>-individually	**Two sessions**<br>-**At baseline,** an average of 30min to complete the session<br>-**At 6mo later** an average of 22min to complete the survey	Online survey company
Petrova 2015<br>Both experiments	Participants in both experiments were randomly assigned to view the statistical information in 1 of 3 formats: text, fact box, or visual aid.	Mortality for men/women (50y or older) from (prostate/breast cancer) with and without screening within 11 years	-No<br>-Participants were provided with background information about prostate or breast cancer and statistical information about the benefits and<br>harms from mammography or PSA test screening before viewing the formats	-NP<br>-Online<br>-individually	One session<br>-average of 15min to complete the survey	Online

Table of interventions

**Author, year**	**Interventions**<br>Brief Name<br>type (graph, text) with details	**Outcome in the methods and time point/period**<br>(ex: survival rate over 10years)	**Co-intervention**<br>(Preceded by training for the participants (yes/no)<br>-involved explanation (yes/no), if yes how and by whom)	**Who**<br>delivered the intervention?<br>If provided (their expertise, background, any specific training given)<br>**How**<br>Mode of delivery (face-to-face, Online, Telephone)<br>Individually or in a group	**When and how much**<br>Over what period, no of sessions, duration	**Where**<br>the intervention was delivered
Zikmund-Fisher 2007	4 versions of Internet-administered survey<br>1- survival curve graph showing 15 years' worth of data **(Survival Graph A)**<br>2- survival graph showing only 5 years' worth of data **(Survival Graph B)**<br>3- mortality curve graph showing 15 years' worth of data **(Survival Graph A)**<br>4- mortality graph showing only 5 years' worth of data **(Survival Graph B)**<br>Also<br>Name of the condition randomly varied<br>-50% read about "Crawford's Disease"<br>-50% read about "Crawford's cancer"<br>- Before randomisation patients read a hypothetical scenario then randomised to 1 of 4 graphs<br>- were not asked to imagine that they had the condition They were informed that patients with this disease have a constant risk of dying each year but can lower that risk by taking one of two medications.	Two graphs showed survival curves indicating the proportion of Crawford's Disease patients who took either Pill A, Pill B, or No Pills still surviving as time since diagnosis increases.<br>The remaining two graphs showed the equivalent mortality data for each option	-No<br>-hypothetical scenario before randomisation	-NR<br>-online<br>-individually	One session<br>Duration NR	Online
Zikmund-Fisher 2008	Participants were viewed a scenario<br>-Each participant was asked to imagine being diagnosed with breast cancer after a routine mammogram.<br>The graphs:<br>1- horizontal stacked bar format used in standard Adjuvant<br>2- graphic included 4 pictographs, 1 for each treatment option, arranged with the no therapy graph on the left and the 3 adjuvant therapy options to the right.<br>**Two simpler graphs** (displayed 2 bars or pictographs rather than 4, two treatment options were only displayed)<br>-all study participants received identical risk information	the mortality risks associated with different adjuvant treatment options over 10 years	-No<br>-A scenario was provided before randomised to interventions (details in page 3 of the article) then viewed the target graphic along with explanatory text.	-NR<br>-online<br>-individually	One session<br>Duration NR	Online
Zikmund-Fischer 2010<br>Primary study and replication study	Participants were viewed a scenario and imagined being diagnosed with breast cancer, Respondents then<br>viewed the target graphic along with explanatory text.<br>1- Multi-outcome pictograph (10x10 matrices, 4color graphic, for both the hormonal therapy–only and the chemotherapy plus hormonal therapy treatment<br>Options)<br>2- Survival-only pictograph (identical to the multi-outcome graphic but 2colors, and only 1 outcome)<br>All study participants received identical risk information. The risk graphic used to display this information was randomly varied among 2 possible choices.	**Multi-outcome pictograph.**<br>-displayed outcomes (survival, incremental survival due to chemotherapy, mortality due to cancer, and other-cause mortality).<br>-Over 10 years<br>**Survival-only pictograph.**<br>-mortality due to cancer and mortality due to other causes.	-No<br>-yes, a vignette (same as Zikmund-Fischer 2008)	-NR<br>-Online<br>-individually	One session<br>Duration NR	Online

Table of outcomes

Author, year	Relevant Outcomes Definition of the outcome	Measurement time points Pre and/or post test	Measurement of the outcome (how the outcome was measured, which tools/surveys/questionnaires used)	Numbers analysed Total n (%) (intervention1, intervention2,..etc)	Follow up rate Intervention 1,2,	Results	Authors' conclusions
Armstrong 2002	1-Understanding of curves<br>2-Treatment choice<br>3-Treatment preferences	Single time point, post intervention	**1- Understanding of curves (6 questions, 2 questions for each type)**<br>**Q1**: How many people who have a colectomy are alive/dead at year 20?<br>**Q2:** In which group are more people alive/dead at year 30?<br>**Q3**: How many more people are alive/dead in this group at year 10?<br>Questions' framing mirrored the format they received<br>Answers were considered accurate only if 2/2 are correct, 1/2 partially accurate, 0/2 inaccurate<br>**2- Treatment choice**<br>by asking participants to decide whether they would want to have a preventive colectomy (i.e., "Given this information, which option would you choose?").<br>**3-Treatment preferences**<br>asked about preference for surgery at 3 levels of benefit of colectomy (5%, 10%, or 20% at 50 years), each shown by a separate graph with 2 curves<br>for all questionnaires, 10% gain was presented first, then 5% and then 20%	-451 (100%)<br>**N**<br>-survival curve 150<br>-mortality curve 151<br>-Both curves 150	**NA**	**1- Understanding (table 2): % answered correctly:**<br>**mortality curve, both curves, survival curve**<br>**Q1:** 54, 67, 69, (p=0.01)<br>**Q2:** 75, 84, 85, (p=0.05)<br>**Q3:** 43, 49, 56, (p=0.02)<br>**All understanding questions**, 38, 48, 52, (p=0.02)<br>**2-Treatment choice**<br>**Choosing preventive surgery**<br>survival curve > both curves > mortality curve<br>**3-Treatment preferences**, at 50 years<br>**mortality curve, both curves, survival curve**<br>5% (39, 51, 55, p=0.02)<br>10% (51, 56, 59, p=0.35)<br>20% (53, 62, 70, p=0.06)	Framing graphic risk information as chance of death overtime results in lower levels of understanding and less interest in preventive surgery than framing as chance of survival over time.

Table of outcomes							
**Author, year**	**Relevant Outcomes Definition of the outcome**	**Measurement time points Pre and/or post test**	**Measurement of the outcome (how the outcome was measured, which tools/surveys/questionnaires used)**	**Numbers analysed Total n (%) (intervention1, intervention2,..etc)**	**Follow up rate Intervention 1,2,**	**Results**	**Authors' conclusions**
Brick 2020	1-Comprehension of harms and benefits<br>2-Treatment decision<br>3-Presentation	**1-Comprehension**<br>Two time points baseline, 6 weeks later<br>**2-Treatment decision**<br>Two time points baseline, 6 weeks later<br>**3-Presenattion**	**1-Comprehension**<br>12 questions: eight were multiple choice questions with five options, and four were open-response requests for numbers.<br>-Each of the 12 items was scored exactly correct or not and a composite of comprehension was calculated from the proportion of correct items.<br>**Q1:** Out of 100 children with a middle ear infection who took antibiotics, how many experienced a ruptured eardrum?<br>**Correct answer: 1**<br>**Q2**: {min=0 max=100} Out of 100 children with a middle ear infection who took placebo, how many continued to have impaired hearing 4-6 weeks after diagnosis?<br>**Correct answer: 40**<br>**Q3:** Children who took which treatment had a higher chance of developing a ruptured eardrum?<br>Antibiotics, both antibiotics and placebo were the same, placebo, this information is not shown, I don't know<br>**Correct answer: placebo**<br>**Q4:** Children who took which treatment had a higher chance of experiencing pain 4-7 days after diagnosis?<br>Antibiotics, both antibiotics and placebo were the same, placebo, this information is not shown, I don't know<br>**Correct answer: placebo**<br>**Q5:** Out of 100 children who received placebo, how many had issues with hearing 4-6 weeks after diagnosis?<br>Almost none, About a quarter, About half, About three quarters, I don't know<br>**Correct answer: about half**<br>**Q6:** which group was less likely to experience pain 4-7 days after diagnosis?<br>Children who took antibiotics, children who took a placebo, the effect was the same in both groups, this information was not shown, I don't know<br>**Correct answer: children who took antibiotic**<br>**2-Treatment decision**<br>**Q7:** The participants were asked whether they would recommend the treatment for a relative. (yes, no, unsure, no difference). Treatment decision is coded as 1 (yes) and 0 (all other responses)<br>**3-Presentation**<br>**Q8:** whether they like how it is presented, rated from 1 (not at all) to 5 (very much)	**Time 1**<br>Ear fact box 599<br>Ear text 576<br>**Time 2**<br>Ear fact box 445<br>Ear text 423<br>**T1/Total number of people answering each Q:**<br>**Fact box, text box**<br>Q1: 593, 569<br>Q2: 593, 568<br>Q3: 593, 569<br>Q4: 593, 569<br>Q5: 593, 569<br>Q6: 593, 567<br>Q7: 593, 568<br>Q8: 2303	1666 (72.3%)<br>Ear fact box 445 (74%)<br>Ear text 423 (73%)<br><br>**T2/Total number of people answering each Q:**<br>**Fact box, text box**<br>Q1: 445, 417<br>Q2: 445, 417<br>Q3: 445, 417<br>Q4: 445, 417<br>Q5: 445, 417<br>Q6: 444, 417<br>Q7: 445, 417<br>Q8: 1666	**1-Comprehension**<br>**T1**<br>**Ear fact box, Ear text box**<br>**n/valid %**<br>Q1: 528/89.1, 501/88.1<br>Q2: 519/87.5, 330/58.2<br>Q3: 515/86.9, 401/70.4<br>Q4: 514/86.6, 440/77.3<br>Q5: 493/83.2, 344/60.5<br>Q6: 465/ 78.4, 423/74.5<br>**T2**<br>**Ear fact box, Ear text box**<br>**n/valid %**<br>Q1: 379/85.1, 326/78.3<br>Q2: 381/85.4, 215/51.6<br>Q3: 379/85.1, 294/70.7<br>Q4: 378/84.8, 326/78.2<br>Q5: 377/84.6, 228/54.7<br>Q6: 351/78.9, 275/66.1<br>**For all comprehension Qs: mean % (SD)**<br>**Ear fact box, ear text**<br>**Time 1:**<br>83.7 (22.7), 73.7 (27.5)<br>**Time 2:**<br>81.6 (25.5), 69.0 (31.1)<br>**Effect size of format on comprehension: Cohen's d=0.39, 95% CI (0.31-0.47)**<br>**2-Treatment decision**<br>**Q7: n/valid %**<br>**T1: fact box** Yes 277/46.7, No 70/11.8, No difference 163/27.5, Unsure 83/14 (total 593)<br>**Text box** Yes 235/41.5, No 95/16.8, No difference 154/27.1, Unsure 84/14.7 (total 568)<br>**T2:**<br>**fact box** Yes 215/48.2, No 57/12.8, No difference 103/23.2, unsure 70/15.8 (total 445)<br>**text box** Yes 159/38.2, No 59/14.2, No difference 120/28.9, unsure 78/18.7 (total 417)<br>**All Q: Ear fact box, ear text** coded as yes=1, other responses=0<br>**mean % (SD)**<br>**Time 1:** 46.9 (49.9), 42.5 (49.5)<br>**Time 2:** 47.0 (49.9), 36.6 (48.2)<br>**3-Presentation**<br>**T1:**<br>Not at all 190/8.2, a little 361/15.7, somewhat 629/27.3, a fair amount 716/31.1, very much 408/17.7<br>**T2:**<br>Not at all 197/11.8, a little 260/15.6, somewhat 503/30.2, a fair amount 474/28.4, very much 232/13.9 (total 1666)	The brief table format of the fact box improved the comprehension of harms and benefits relative to the text-only control across diverse levels of education and numeracy.

Table of outcomes

Author, year	Relevant Outcomes Definition of the outcome	Measurement time points Pre and/or post test	Measurement of the outcome (how the outcome was measured, which tools/surveys/questionnaires used)	Numbers analysed Total n (%) (intervention1, intervention2,..etc)	Follow up rate Intervention 1,2,	Results	Authors' conclusions
Hamstra 2015 25277673	1-Verbatim knowledge: "the ability to understand the exact numbers presented."<br><br>2-Graphical preference and expected understanding	Postintervention	**1- Knowledge was assessed with 4 questions, a correct answer as that ±1 from the number presented.**<br>**Q1:** How many people will have their prostate cancer come back in year 1 if they did not use hormone therapy?<br>**Q2:** How many people will have their prostate cancer come back in year 2 if they did not use hormone therapy?<br>**Q3:** For people who take hormone therapy, how many people will have their prostate cancer come back in year 1?<br>**Q4:** For people who take hormone therapy, how many people will have their prostate cancer come back in year 3?<br>**2- Graphical preference was measured with 2 questions, (participants were viewed all formats at this stage)**<br>**Q5:** If you had to make an important medical decision and you wanted to learn about its risks and benefits of treatment, which of these ways of presenting the information would you prefer to receive?<br>**Q6:** If you had to make an important medical decision and you wanted to learn about its risks and benefits of treatment, which of these ways of presenting the information would you understand better?<br><br>**N.B rating about graph experience was not extracted**	**Total 420**<br>**F1:** Bar graph 1 (54)<br>**F2:** Bar graph 2 (51)<br>**F3:** Line graph 1 (56)<br>**F4:** Pie graph 1 (50)<br>**F5:** Pie graph 2 (54)<br>**F6:** Pictograph (56)<br>**F7:** Pictograph 1 (49)<br>**F8:** Pictograph 2 (50)	**NA**	**1-Knowledge**<br>**F1, F2, F3, F4, F5, F6, F7, F8, *P* value (Adjusted Wald X^2)**<br>**Q1:** 91, 88, 86, 84, 89, 88, 82, 92, 0.46<br>**Q2:** 87, 86, 71, 82, 89, 84, 88, 90, 0.28<br>**Q3:** 87, 92, 80, 90, 87, 89, 88, 94, 0.49<br>**Q4:** 83, 84, 79, 86, 85, 91, 90, 92, 0.66<br>**Overall % correct:** 87, 88, 79, 86, 88, 88, 87, 92, 0.35<br><br>**Q1, Q2, Q3, Q4, overall % correct**<br>**Wald X^2 P value:** 0.81, 0.20, 0.56, 0.53, 0.55<br>**Adjusted Wald X^2 P value:** 0.46, 0.28, 0.49, 0.66, 0.35<br><br>Comparing graph types by overall knowledge score, graphs with 2 numbers resulted in marginally higher knowledge (OR =1.3; CI=0.9-2.5; *P* =0.10).<br>However, there was no statistically significant difference between graph types (F2, F5, F8). Similarly, there was no difference among graphs with 1 number (F1, F3, F4, F7). Pictographs with 2 numbers demonstrated the best verbatim; answering correctly 92% (±3% SEM) of the time while line graph with 1 number was the poorest answering correctly 79% (±4% SEM) of the time (*P*=0.018)<br>Comparing only pictograph formats: the increase of numbers has statistically significant effect: F6 88% (±4% SEM), F7 87% (±3% SEM), F8 92% (±3% SEM) **Graphical preference**<br>**Q5: % choosing**<br>Bar graph 31, Pie graph 27, Line graph 25, Pictograph 10<br>**Q6: % choosing**<br>Bar graph 29, Pie graph 26, Line graph 27, Pictograph 10	Pictographs were one of the most effective means of transferring information regardless of numeracy.
Kasper 2017	1-comprehension of the given quantitative information.	immediately after display of graphical presentation	measure of accurate comprehension of the given quantitative information.<br>4 questions of comprehension **(provided in the table)**<br>**The only eligible Q**<br>**Q:** How many of 100 patients remain stable without interferon?<br>**Correct answer:71**<br>The score was dichotomized, defining four correct answers to the given set of four questions as correct and any other combination as false including missing answers	**Total 682**<br>**Gp1:** MFP-static **(**193)<br>**Gp2:** MFP-animated **(**171)<br>**Gp3:** CLARIFIG static **(**160)<br>**Gp4**: CLARIFIG animated **(**158)	**General dropout rate**<br>(n=24, 2.7%)<br>**Lost to follow up**<br>**Gp1:** 4 (2.1%)<br>**Gp2:** 11 (6.4%)<br>**Gp3:** 7 (4.4%)<br>**Gp4:** 2 (1.3%)	**Results in the four-item comprehension test**<br>**Gp1, Gp2, Gp3, Gp4**<br>**N =** 193, 171, 160, 158<br>**Q3:** 86%, 39%, 91%, 90%<br>**Total score** 88 (46%), 39 (23%), 68 (44%), 48 (30%)<br>Gp1 (46%)/Gp3 (44%)→ *P*=0.59 (all questions)<br>Gp2 (23%)/Gp4 (30%)→ *P*=0.134 (all questions)<br>Generally: The two formats did not differ with regard to frequencies of<br>comprehension, neither in the static nor in the animated presentation<br>compared to the static presentation, **the animated formats led to significantly less comprehension and longer processing time** (Table 3).<br>Numeracy was positively related to comprehension (P=.016)	Using the new and condensed format CLARIFIG, the quantitative information can be presented as understandably as using the well-established MFPs

Table of outcomes

<table>
<tr><th>Author, year</th><th>Relevant Outcomes Definition of the outcome</th><th>Measurement time points Pre and/or post test</th><th>Measurement of the outcome (how the outcome was measured, which tools/surveys/questionnaires used)</th><th>Numbers analysed Total n (%) (intervention1, intervention2,..etc)</th><th>Follow up rate Intervention 1,2,</th><th>Results</th><th>Authors' conclusions</th></tr>
<tr><td>McDowell 2019</td><td>Study 1
1-Comprehension
2-Decision intentions</td><td>1-Comprehension
-assessed at baseline (while presenting the formats),
-shortly after presentation (7min delay) for knowledge recall (ineligible for our SR)
2-Decisional intentions
While viewing the format
Short delay (7min delay)</td><td>1-Comprehension (some questions are not eligible, results for these Qs are not extracted)
Assessed using 8 Qs; 3 Qs assessed knowledge of the benefit of screening and 5 Qs assessed knowledge of harms of screening, 2 questions were eligible
Q1 How many men who participate in screening will die from prostate cancer over 10 years? out of 1000
Q2 How many men who do not participate in screening will die from prostate cancer over 10 years? out of 1000
Numerical estimates were coded as correct within a ±10-point margin of error
2-Decision intentions
Q3 Participants were asked whether they planned to participate in prostate cancer screening in the next 6 months and, if not, to indicate the most important reason for their response</td><td>1-Comprehension N
F1: Tabular format (40)
F2: Single icon array (39)
F3: Separate icon array (41)
All the people randomised were analysed
2- Decision intentions
In the results table, see also footnote</td><td>NA</td><td>1- Comprehension (Formats Were Presented Alongside Questions)
% correct: F1, F2, F3
Q1: 77.5, 87.2, 75.6
Q2: 70, 82.1, 73.2
Mean of all questions 5.8 out of 8
-There were no statistical differences between the fact box formats.
- > 90% of all participants correctly reported that the same number of men die from prostate cancer in screened and non-screened groups at both assessment points, irrespective of whether or not the actual numbers were correct.
2-Decion intention [1]
<table>
<tr><td></td><td>Intension to screen</td><td>Yes N/%</td><td>No N/%</td></tr>
<tr><td>F1</td><td>While viewing format</td><td>13/32.5</td><td>27/22.5</td></tr>
<tr><td></td><td>Short delay</td><td>11/27.5</td><td>29/24.1</td></tr>
<tr><td>F2</td><td>While viewing format</td><td>16/41</td><td>23/19.1</td></tr>
<tr><td></td><td>Short delay</td><td>14/35.9</td><td>25/20.8</td></tr>
<tr><td>F3</td><td>While viewing format</td><td>10/24.4</td><td>31/25.8</td></tr>
<tr><td></td><td>Short delay</td><td>9/22</td><td>32/26.6</td></tr>
<tr><td>Total</td><td>While viewing format</td><td>39/32.5</td><td>81/67.5</td></tr>
<tr><td></td><td>Short delay</td><td>34/28.3</td><td>86/71.6</td></tr>
</table>
While viewing the format, after short delay %
Intended to screen 33, 28
Not 37, 37
No answer 31, 35
There were no differences in decision intentions between formats at any assessment point.
Responses were largely unchanged after the short delay</td><td>Fact box formats (with or without icon arrays) were equally effective at facilitating comprehension and short-term knowledge recall and led to similar decision intentions and perceptions of having complete information about screening to make a decision.

Separate icon arrays improved comprehension of nonnumerical items, whereas single icon array facilitated answering numerical questions</td></tr>
</table>

[1] As the number of participants who did not indicate an intention to screen changed at each assessment point, the samples contained different participants. Numbers do not add up to 100%; participants who indicated they had other reasons for not intending to participate in prostate cancer screening are not shown. Only these 2 categories were provided to participants in study 1.

Table of outcomes

<table>
<tr><th>Author, year</th><th>Relevant Outcomes Definition of the outcome</th><th>Measurement time points Pre and/or post test</th><th>Measurement of the outcome (how the outcome was measured, which tools/surveys/questionnaires used)</th><th>Numbers analysed Total n (%) (intervention1, intervention2,..etc)</th><th>Follow up rate Intervention 1,2,</th><th>Results</th><th>Authors' conclusions</th></tr>
<tr>
<td>McDowell 2019[2]</td>
<td>Study 2
1-Comprehension
2-Decision intentions
3-perceived risk</td>
<td>1-Comprehension
-assessed at baseline (while presenting the formats),
-shortly after presentation (5min delay) for knowledge recall (ineligible for SR)
-6months later
2-Decisional intentions
While viewing the format, short delay (7min)</td>
<td>1,2,3 same as previous

2-Decision intentions
In addition, participants who did not indicate an intention to screen were provided with additional answer options to try and categorize ''other'' reasons against screening.
3- perceived risk
assessed by asking participants to consider 100 men like themselves and indicate how many they think would die from prostate cancer within the next 10 y.</td>
<td>1-knowledge N
Total analysed 225
F1: Tabular format (47)
F2: Single icon array (58)
F3: Separate icon array (56)
Standard information 64[3]

After 6 months
Total analysed 143
Tabular format (F1) 30
Single icon array (F2) 39
Separate icon array (F3) 34
Standard information 40</td>
<td>152 completed the follow up (out of 244 at baseline)</td>
<td>1-Comprehension (Formats Were Presented Alongside Questions)
% correct:
F1, F2, F3 (comprehension at 6mon)
Q1: 80, 64, 56
Q2: 70, 62, 56

2-Decion intention
<table>
<tr><td></td><td>F1</td><td>F1</td><td>F2</td><td>F2</td><td>F3</td><td>F3</td></tr>
<tr><td>Intension to screen</td><td>Yes N/%</td><td>No N</td><td>Yes N/%</td><td>No N</td><td>Yes N/%</td><td>No N</td></tr>
<tr><td>Baseline</td><td>11/23.4</td><td>36</td><td>15/25.9</td><td>43</td><td>19/33.9</td><td>37</td></tr>
<tr><td>Post</td><td>11/23.4</td><td>36</td><td>15/25.9</td><td>43</td><td>13/23.2</td><td>43</td></tr>
<tr><td>6mo</td><td>6/20</td><td>24</td><td>6/15.4</td><td>33</td><td>12/35.3</td><td>22</td></tr>
<tr><td>While viewing the format</td><td>5/16.7</td><td>25</td><td>6/15.4</td><td>33</td><td>7/20.6</td><td>27</td></tr>
</table>
While viewing the format, after short delay % (all formats)
Intended to screen 33, 28/ Not 37, 37/ No answer 31, 35
There
3-Risk perception:
17.1% provided the answer (7 out of 100)/ actual 0.7</td>
<td>Similar to study 1 and in addition to that:
Fact boxes are an efficient means to improve comprehension at the time of presentation, as well as short-term recall but not long-term recall.

Fact boxes improved both knowledge recall and comprehension, whereas there were no improvements when participants received widely available cancer health information.</td>
</tr>
</table>

[2] Only comprehension at 6 months was extracted here

[3] Standard format data was not added here as not eligible to our systematic review (reason: it contains different information than other formats)

Table of outcomes							
**Author, year**	**Relevant Outcomes Definition of the outcome**	**Measurement time points Pre and/or post test**	**Measurement of the outcome (how the outcome was measured, which tools/surveys/questionnaires used)**	**Numbers analysed Total n (%) (intervention1, intervention2,..etc)**	**Follow up rate Intervention 1,2,**	**Results**	**Authors' conclusions**
Petrova 2015<br>Experiment 1	1-Comprehension<br>2-Intentions<br>a-intention to be screened<br>b-intention to look for information about prostate/breast cancer screening.	Postintervention	**1-Comprehension**<br>**Assessed using** 8 questions<br>**The eligible questions Q6,7**<br>**Q1** If 2,000 men (women) participate in screening, how many men (women) will die from prostate (breast) cancer in the next 11(10) years?<br>**Correct answer: 14**<br>**Q2** If 2,000 men (women) do NOT participate in screening, how many men (women) will die from prostate (breast) cancer in the next 11(10) years?<br>**Correct answer: 14**<br>**2-Intentions**<br>participants indicated on scales from 1 (absolutely disagree) to 7 (absolutely agree), what extent they agreed with the following statements:<br>a- Intend to participate in prostate/breast cancer screening<br>b- Intend to look for more information about prostate/breast cancer screening.	Number of people answering each Q was NR	NA	**1-Comprehension**<br>**Comprehension (mean of score 0-8/SD)**<br>Experiment 1 (5.23/2.04)<br>**% correct**<br>**Experiment 1**<br>Q1 78%, Q2 78%<br>**% correct per format (for all comprehension Qs)**<br>Visual aids 72, Textual message 64, Fact box 61<br>Generally: The information format had a significant effect on comprehension.<br>**2-Intentions**<br>**Experiment 1 (screening statistics showed no benefits but showed substantial harm)**<br>**(mean/SD)** (4.21/ 2.00)<br>44% of participants intended to participate in screening (a score>4)<br>37% intended not to participate (a score<4)<br>**Experiment 2 (When screening had both benefits and harms)**<br>**(mean/SD)** (5.12/ 1.94)<br>66% intended to participate (score>4)<br>21% intended not to participate (score<4)<br>**significance** p<br>format on comprehension effect 0.018<br>visual/text 0.05 visual better than text<br>visual/fact box 0.003 visual better than fact box<br>fact box/text 0.314 no difference	Visual aids increased comprehension compared to textual message and fact box. The fact box was not significantly different to the textual format

Table of outcomes							
**Author, year**	**Relevant Outcomes Definition of the outcome**	**Measurement time points Pre and/or post test**	**Measurement of the outcome (how the outcome was measured, which tools/surveys/questionnaires used)**	**Numbers analysed Total n (%) (intervention1, intervention2,..etc)**	**Follow up rate Intervention 1,2,**	**Results**	**Authors' conclusions**
Petrova 2015<br>Experiment 2	Same as above	Same as above	Same as above	Same as above	Same as above	**1-Comprehension**<br>**Comprehension (mean of score 0-8/SD)**<br>Experiment 2 (4.81/1.76)<br>**Experiment 2**<br>Q1 70%, Q2 74%<br>**% correct per format (for all comprehension Qs)**<br>**Women who perceived breast cancer to be moderately severe**<br>Visual aids 67, Textual message 61, Fact box 60<br>**Women who perceived breast cancer to be extremely severe**<br>Visual aids 57, Textual message 63, Fact box 55<br>**Generally**<br>On average, participants in both experiments correctly answered 62% (95% CI, 60%-64%) of the comprehension questions,<br>65% [95% CI, 62%-69%] in experiment 1<br>60% [95% CI, 58%-62%] in experiment 2.<br>The information format had no significant main effect on comprehension<br>**2-Intentions**<br>**Experiment 2 (When screening had both benefits and harms) (mean/SD)** (5.12/ 1.94)<br>66% intended to participate (score>4)<br>21% intended not to participate (score<4)<br>**significance** p<br>format on comprehension effect 0.281<br>moderate severity visual/other formats 0.053<br>extreme severity text/other formats 0.02	Visual aids increased comprehension among women who perceived breast cancer to be moderate<br><br>Textual message was the best format to increase comprehension for women who perceived breast cancer as extremely severe

Table of outcomes

Author, year	Relevant Outcomes Definition of the outcome	Measurement time points Pre and/or post test	Measurement of the outcome (how the outcome was measured, which tools/surveys/questionnaires used)	Numbers analysed Total n (%) (intervention1, intervention2,..etc)	Follow up rate Intervention 1,2,	Results	Authors' conclusions
Zikmund-Fischer 2007	1-Comprehension of the graphs	Postintervention	**1-Comprehension:**<br>**Using a sequence of comprehension questions about the information presented in the graph**<br>**Q1:** which pill was more effective? Pill A/Pill B<br>**Q2:** how many people who took Pill A, out of 100, were dead after 12 years (4 years if respondents saw the abbreviated 5-year ''B'' graphs)<br>**Q3:** No. who took No Pills dead after 3/9 years?<br>**Q4:** No. who took Pill B alive after 5/15 years?<br>**Q5:** No. who took Pill B who died between 1 and 5 years/between 5 and 15 years?<br>Responses were coded as correctly answered if they were within ±4 people of the true answer.<br>**Q6:** If someone survived 3/9 years, which pill gives the best chance of surviving another year?	Survival graphs (n=710)<br>Mortality graphs (n=751)	Dropout from the study n=243,14%	**1- Comprehension questions**<br>**% Correctly answering (Among people correctly identifying Pill A as more effective than Pill B),**<br>**Survival graphs, Mortality graphs, significance (p)**<br>**Q1** 93.9, 84.8, <0.001<br>**Q2** 61.5, 80.1, <0.001<br>**Q3** 54.4,72.7, <0.001<br>**Q4** 69.0, 72.7, 0.163<br>**Q5** 37.5,38.1, 0.835<br>**Q6** 95.7, 91.5, 0.004	The number of years of data provided in a survival curve can change beliefs about treatment effectiveness by itself. Presenting data in mortality graph format significantly reduces this unwanted effect, although special care will need to be taken to ensure that readers of mortality graphs recognize that the optimal treatment is shown by the lowest curve.<br>All presentations of risk graphics (whether framed in survival or mortality terms) highlight duration information to facilitate comprehension.
Zikmund-Fisher 2008	1-knowledge of the risk statistics<br>2-preference ratings for the graph	Post intervention	**1-assessed with 3 questions:**<br>**Q1:** the chance that the respondent would be alive in 10 years with hormonal therapy only<br>**Q2:** the chance the respondent would be alive with both chemotherapy and hormonal therapy<br>**Q3:** how many fewer women out of 100 would die from cancer if they received both chemotherapy and hormonal therapy instead of hormonal therapy only<br>Correct answer to Q3: 2 out of 100<br>-Exact numerical information sufficient to calculate these answers was provided in the graph legends, responses were only coded as accurate when they were exactly correct.<br>**2-preference ratings for the graph**<br>Answering each question on a 10-point scale, respondents rated how well the graph described the benefits of different additional treatments, whether the respondent would prefer to see risk information in this type of graph, and how clearly the graphs represented the increase in the chance of being alive.	Data available for analysis (n=1619)<br>**Total number of people answered each Q**<br>**4-Option Graph**<br>Horizontal bar<br>Q1 393, Q2 401<br>Pictograph<br>Q1 389, Q2 405<br>**2-Option Graph**<br>Horizontal bar<br>Q1 405, Q2 410<br>Pictograph<br>Q1 364, Q2 378<br><br>**Numbers were not provided for Q3**	NA	**1- knowledge of the risk statistics**<br>**% correctly reporting total survival rate by graph type**<br>**Q1: 4-option graph** (horizontal bar, pictograph) 17.6, 33.4<br>**2-Option Graph** (horizontal bar, pictograph) 65.9, 64.3<br>**Q2: 4-option graph** (horizontal bar, pictograph) 16.7, 31.6<br>**2-Option Graph** (horizontal bar, pictograph) 37.3, 49.7<br>**Q3: 4-option graph** (horizontal bar, pictograph) 51.1,53.7<br>**2-Option Graph** (horizontal bar, pictograph) 64.6, 77.2<br>**Significance all versus 4-option horizontal bar graph (all significant) (chi-square)**<br>**4-option pictograph, 2-option horizontal bar graph, 2-option pictograph**<br>**Q1:** 25.93, 191.43, 171.89<br>**Q2:** 24.38, 43.56, 96.39<br>**2- Preference ratings for the graph**<br>respondents showed strong preferences for **pictograph formats** over the currently used horizontal bar format.<br>Participants who viewed the 2-option pictographs took the least amount of time to complete knowledge tasks (Fig. 5) and had the lowest error rates (fig 4)	2-option pictographs had higher comprehension rates than<br>those who viewed 4-option bar graphs, regardless of numeracy score

Table of outcomes

Author, year	Relevant Outcomes Definition of the outcome	Measurement time points Pre and/or post test	Measurement of the outcome (how the outcome was measured, which tools/surveys/questionnaires used)	Numbers analysed Total n (%) (intervention1, intervention2,..etc)	Follow up rate Intervention 1,2,	Results	Authors' conclusions
Zikmund-Fischer 2010<br>Primary study	1- Comprehension.<br>2-Graph evaluation ratings<br>3-Treatment intentions.	Post intervention	**1- Comprehension**<br>**using 3 questions**<br>**Q1:** the chance that the respondent would be alive in 10 y with hormonal therapy only<br>**Q2:** the chance she would be alive with both chemotherapy and hormonal therapy<br>**Q3:** how many fewer women out of 100 would die from cancer if they took both chemotherapy and hormonal therapy instead of hormonal therapy only<br>**responses were coded as accurate only if exactly correct**<br>**2-Graph evaluation ratings**<br>**using 3 questions**<br>**Q4:** how well the graph described the benefits of different additional<br>Treatments<br>**Q5:** whether the respondent would prefer to see risk information in this type of graph<br>**Q6:** how clearly the graphs represented the increase in the chance of being alive<br>Respondents answered each question using a 10-point scale.<br>**3-Treatment intentions**<br>how likely do you think you would be to take both chemotherapy and hormonal therapy?<br>On a 10-point scale with endpoints labelled as extremely likely NOT to take chemotherapy and extremely likely to TAKE chemotherapy	Total 838<br>**Total number of people answered each Q**<br>**Multi-outcome graph**<br>Q1: 364<br>Q2: 378<br>Q3: 381<br>**Survival only graph**<br>Q1: 436<br>Q2: 448<br>Q3: 450	NA	**1- Comprehension**<br>**Multi-outcome Graph, Survival-Only Graph <u>table 2</u>**<br>**N (%) correct**<br>**Q1:** 234 (64.3), 308 (70.6)<br>survival-only graph elicited marginally better comprehension<br>**Q2:** 188 (49.7), 281 (62.7)<br>Compared with the multi-outcome graph, the survival-only graph elicited significantly better comprehension<br>**Q3**: 294 (77.2), 355 (78.9)<br>women presented with the simpler survival-only graphics had comprehension that was as good as, and sometimes better than, women who viewed the more complicated multi-outcome graphic.<br>**<u>test of significance</u>**<br>X^2 p<br>Q1: 14.09 <0.001<br>Q2: 14.09 <0.001<br>Q3: 0.36 0.55<br>**2-Graph evaluation ratings**<br>**Q4-6 combined as were highly correlated**<br>Survival-only pictographs were rated significantly better than multi-outcome<br>pictographs mean (s) rating: 7.98 (2.34) versus 7.68 (2.08), t =2:01, P =0:04.<br>Education was not significantly associated with evaluation ratings.<br>**3-Treatment intentions**<br>**% choosing chemotherapy fig4**<br>**Multi-outcome Graph, Survival-Only Graph** 50.3%, 43.1%, p=0.04<br>-Participants who viewed the survival-only graphic were significantly less likely to say that they preferred adding chemotherapy to hormonal therapy (P =0:04).<br>-More numerate individuals were less likely to select chemotherapy (OR = 0.87, P =0:04)<br>-no significant interactions between survival-only format and either respondent numeracy or education.	Taking a ''less is more'' approach by omitting redundant mortality outcome statistics can be an effective method of risk communication and may be preferable when using visual formats such as pictographs.
Zikmund-Fischer 2010<br>Replication study	Same as the primary		**Same as the primary except for 3- treatment intentions**<br>**Assessed by asking**: ''At this point, would you want to take both chemotherapy and hormonal therapy or hormonal therapy only?''	714 participants	NA	**1-Comprehension**<br>-Survival-only graph had better comprehension of the total number of women alive with combined therapy than participants who viewed multi-outcome graph, although the difference did not reach statistical significance (survival-only: 64% v. multi-outcome: 58%, P =0:13).<br>**2-treatment intention**<br>- Intentions to take chemotherapy were lower in the survival-only group (21% v. 27%, P =0:06, in bivariate analysis; OR=0.67, P =0:03, in multivariate logistic regression).<br>- Respondents with higher numeracy scores chose chemotherapy less often (OR=0.69, P <0:001).<br>- Participants provided higher graph evaluation ratings for survival-only graphs than for multi-outcome graphs (x (s) rating: 8.06 (2.21) v. 7.72 (2.32), t =2:05, P =0:04).	

Table of the excluded studies

	Author	Year	Reason category for exclusion	Reason
1.	Abdul, R. M., et al.	2019	Ineligible intervention	Report about prediction model
2.	Stiggelbout, A. M., et al.	2008	Ineligible outcome	Comprehension was not measured
3.	Adarkwah, C. C., et al.	2019	Ineligible outcome	Comprehension was not measured
4.	Adarkwah, C. C., et al.	2016	Ineligible outcome	Comprehension was not measured
5.	Armstrong, K., et al.	2001	Ineligible intervention	Not compared to other interventions
6.	Brundage, M., et al.	2005	Ineligible study design/ type	Qualitative study design
7.	Barnes, C. S., et al.	2009	Ineligible intervention	Compared to usual care
8.	Barry, M. J., et al.	1997	Ineligible intervention	The interventions compared different information (not different visual presentations)
9.	Baumann, B. C., et al.	2018	Ineligible intervention	Not compared to other interventions
10.	Belkora, J. K., et al.	2011	Ineligible intervention	Compared to usual care
11.	Braeken, A., et al.	2014	Ineligible intervention	Not compared to other interventions
12.	Brewer, N. T., et al.	2012	others	Unclear measurement of the outcome, the author could not provide the data needed
13.	Brewer, N. T., et al.	2009	Ineligible outcome	Unclear measurement of the outcome
14.	Brito, J. P., et al.	2015	Ineligible intervention	Compared to usual care
15.	Brower, M. E., et al.	2009	Ineligible outcome	Qualitative measurement of the outcome
16.	Farwati, M., et al.	2018	Ineligible study design/ type	Qualitative study design
17.	Guillen, U., et al.	2016	Ineligible study design/ type	Qualitative study design
18.	Hakone, A., et al.	2017	Ineligible study design/ type	Qualitative study design
19.	Adarkwah, C. C., et al.	2017	Ineligible outcome	Comprehension was not measured
20.	Chang, M. C.	2015	Ineligible intervention	Report about prediction model
21.	Carling, C. L. L., et al.	2009	Ineligible outcome	Comprehension was not measured
22.	Mazur, D. J., et al.	1999	Ineligible study design/ type	Qualitative study design
23.	Cox, C. E., et al.	2011	Ineligible intervention	Compared to usual care
24.	Cox, C. E., et al.	2012	Ineligible intervention	Compared to usual care
25.	Cox, C. E., et al.	2019	Ineligible intervention	Compared to usual care
26.	Coylewright, M., et al.	2016	Ineligible intervention	Compared to usual care

27.	Declercq, E.	2013	Ineligible study design/ type	Debate study, review of literature and examination of data
28.	El-Jawahri, A., et al.	2015	Ineligible intervention	Compared to usual care
29.	Farwati, M., et al.	2018	Ineligible study design/ type	A meeting abstract
30.	Feldman-Stewart, D., et al.	2007	Ineligible intervention	Unclear about the provided data in the intervention
31.	Pohlmann-Eden, B., et al.	2019	Ineligible study design/ type	Qualitative study design
32.	van Maurik, I. S., et al.	2019	Ineligible study design/ type	Qualitative study design
33.	Fortnum, D., et al.	2015	Ineligible study design/ type	A report
34.	Flynn, D., et al.	2015	Ineligible outcome	Qualitative measurement of the outcome
35.	Knops, A. M., et al.	2014	Ineligible outcome	Qualitative measurement of the outcome
36.	Woodhead, E. L.	2009	Ineligible outcome	Qualitative measurement of the outcome
37.	Sugar, E., et al.	2013	Ineligible intervention	Not about prognosis of a health condition
38.	Armstrong, K., et al.	2005	Ineligible intervention	Not about prognosis of a health condition
39.	Heesen, C., et al.	2007	Ineligible intervention	Not compared to other interventions
40.	Heesen, C., et al.	2007	Ineligible study design/ type	A report
41.	Bruine de Bruin, W. and K. G. Carman	2012	Ineligible intervention	Not about prognosis of a health condition
42.	Hoffmann, T., et al.	2007	Ineligible intervention	comparing mode of delivering the information
43.	Bruine de Bruin, W. and K. G. Carman	2018	Ineligible intervention	Not about prognosis of a health condition
44.	Hutton, D. W., et al.	2009	Ineligible intervention	Not compared to other interventions
45.	Clarke, M. G., et al.	2008	Ineligible intervention	Not about prognosis of a health condition
46.	Isebaert, S., et al.	2007	Ineligible intervention	Not compared to other interventions
47.	Jadoon, N. A., et al.	2014	Ineligible intervention	No method of presenting prognostic information was used
48.	Janz, N. K., et al.	2017	Ineligible intervention	No method of presenting prognostic information was used
49.	Chapman, A. R., et al.	2015	Ineligible outcome	Comprehension was not measured
50.	Fraenkel, L., et al.	2016	Ineligible intervention	Not about prognosis of a health condition
51.	Johnson, B. R., et al.	2006	Ineligible intervention	Compared to usual care
52.	Jaspers, N. E. M., et al.	2019	Ineligible outcome	Comprehension was not measured

53.	Gopalan, A., et al.	2014	Ineligible intervention	Not about prognosis of a health condition
54.	Kang, S. K., et al.	2018	Ineligible intervention	No specific time period in the intervention
55.	Juraskova, I., et al.	2009	Ineligible outcome	Comprehension was not measured
56.	Kasper, J., et al.	2006	Ineligible study design/ type	before-after study design
57.	Kasper, J., et al.	2014	Ineligible study design/ type	Abstract, related to included study (Kasper 2017)
58.	Klaassen, L., et al.	2017	Ineligible study design/ type	before-after study design
59.	Han, P. K., et al.	2012	Ineligible intervention	Not about prognosis of a health condition
60.	Hembroff, L. A., et al.	2004	Ineligible intervention	Not about prognosis of a health condition
61.	Krishnamoorthi, R., et al.	2016	Ineligible study design/ type	Evidence synthesis not a primary study
62.	Hong, C., et al.	2013	Ineligible intervention	Not about prognosis of a health condition
63.	Lam, J., et al.	2016	Ineligible intervention	Not compared to other interventions
64.	Irwin, E., et al.	1999	Ineligible intervention	Not about prognosis of a health condition
65.	Leiter, N., et al.	2018	Ineligible intervention	No method of presenting prognostic information was used
66.	Lipkus, I. M., et al.	2010	Ineligible study design/ type	before-after study design
67.	Karns, T. E.	2014	Ineligible outcome	Comprehension was not measured
68.	Jesin, J., et al.	2018	Ineligible intervention	Not about prognosis of a health condition
69.	Lobb, E. A., et al.	1999	Ineligible outcome	Comprehension was not measured
70.	Martin, R. W., et al.	2017	Ineligible intervention	No specific time period in the intervention
71.	Martin, R. W., et al.	2012	Ineligible outcome	Comprehension was not measured
72.	Kakkilaya, V., et al.	2011	Ineligible intervention	Not about prognosis of a health condition
73.	Mazur, D. J. and D. H. Hickam	1994	Ineligible outcome	Comprehension was not measured
74.	McBride, E., et al.	2016	Ineligible intervention	Not compared to other interventions
75.	Kortel, et al.	2017	Ineligible intervention	Not about prognosis of a health condition
76.	Kupke, J., et al.	2012	Ineligible intervention	Not about prognosis of a health condition
77.	Morony, S., et al.	2017	Ineligible study design/ type	A review of materials
78.	Moulton, H., et al.	2018	Ineligible study design/ type	before-after study design

79.	Mazur, D. J. and J. F. Merz	1994	Ineligible outcome	Comprehension was not measured
80.	Murray, E., et al.	2001	Ineligible intervention	Compared to usual care
81.	Murray, G. D., et al.	2018	Ineligible intervention	Not compared to other interventions
82.	Nakano, K., et al.	2018	Ineligible outcome	Comprehension was not measured
83.	Oostendorp, L. J. M., et al.	2017	Ineligible intervention	Compared to usual care
84.	Zamarian, L., et al.	2013	Ineligible outcome	Comprehension was not measured
85.	Pablo Brito, J., et al.	2015	Ineligible study design/ type	Abstract of interim report
86.	Patanwala, I., et al.	2009	Ineligible intervention	The interventions compared different information (not different visual presentations)
87.	Patterson, R., et al.	2015	Ineligible intervention	Not compared to other interventions
88.	Patzer, R. E., et al.	2018	Ineligible intervention	Not compared to other interventions
89.	Perestelo-Perez, L., et al.	2016	Ineligible intervention	Compared to usual care
90.	LeBlanc, A., et al.	2015	Ineligible intervention	Not about prognosis of a health condition
91.	Losina, E., et al.	2017	Ineligible intervention	Not about prognosis of a health condition
92.	Zimmermann, C., et al.	2000	Ineligible outcome	Comprehension was not measured
93.	Mietlowski, W. L., et al.	2013	Ineligible intervention	Not about prognosis of a health condition
94.	Moll, J. M.	1986	Ineligible intervention	Not about prognosis of a health condition
95.	Rolison, J. J., et al.	2016	Ineligible outcome	Unusable data
96.	Prado, M. G., et al.	2016	Ineligible intervention	Not about prognosis of a health condition
97.	Scott, G., et al.	2016	Ineligible intervention	Not compared to other interventions
98.	Rakow, T., et al.	2012	Ineligible intervention	Not about prognosis of a health condition
99.	Rakow, T., et al.	2015	Ineligible intervention	Not about prognosis of a health condition
100.	Schwartz, L. M., et al.	2009	Ineligible intervention	Not comparing different visual presentations
101.	Sears, S. R., et al.	2007	Ineligible intervention	Not comparing different visual presentations
102.	Sheridan, S. L., et al.	2010	Ineligible intervention	Not about prognosis of a health condition
103.	Skibo, M., et al.	2017	Ineligible intervention	Not compared to other interventions
104.	Stephan, L. S., et al.	2018	Ineligible intervention	Not compared to other interventions
105.	Moyer, A., et al.	2017	Ineligible population	Health professionals or students of a health degree

106.	Ozanne, E. M., et al.	2015	Ineligible population	Health professionals or students of a health degree
107.	Sheridan, S. L., et al.	2003	Ineligible intervention	Not about prognosis of a health condition
108.	Silver, B., et al.	2012	Ineligible intervention	Not about prognosis of a health condition
109.	Silver, E. P., et al.	2019	Ineligible intervention	Not comparing different visual presentations
110.	Tait, A. R., et al.	2010	Ineligible intervention	Not about prognosis of a health condition
111.	Vol, et al.	2012	Ineligible intervention	Not about prognosis of a health condition
112.	Watts, K. J., et al.	2014	Ineligible intervention	The two groups received different information
113.	Wenzel, L. B., et al.	2017	Ineligible intervention	Not compared to other interventions
114.	Price, M., et al.	2007	Ineligible population	Health professionals or students of a health degree
115.	Waters, E. A., et al.	2007	Ineligible intervention	Not about prognosis of a health condition
116.	Witteman, H. O., et al.	2014	Ineligible intervention	Not about prognosis of a health condition
117.	Wolpin, S., et al.	2015	Ineligible intervention	Not compared to other interventions
118.	Woloshin, S. and L. M. Schwartz	2011	Ineligible intervention	Not comparing different visual presentations
119.	Yun, Y. H., et al.	2011	Ineligible intervention	The interventions compared different information (not different visual presentations)
120.	Studts, J. L., et al.	2005	Ineligible population	Health professionals or students of a health degree
121.	Zeng-Treitler, Q., et al.	2016	Ineligible intervention	Not about prognosis of a health condition
122.	Zikmund-Fisher, B. J.	2014	Ineligible study design/ type	Viewpoint
123.	Zikmund-Fisher, B. J., et al.	2011	Ineligible intervention	Not comparing different visual presentations
124.	Whelan, T., et al.	2004	Ineligible population	Health professionals or students of a health degree
125.	Bansback	2016	Ineligible intervention	No specific time period in the intervention
126.	Barnes,	2016	Ineligible intervention	Not about prognosis of a health condition
127.	Brewer,	2012	Ineligible intervention	Not about prognosis of a health condition
128.	Brown,	2011	Ineligible intervention	Not about prognosis of a health condition
129.	Chao,	2003	Ineligible population	Health professionals or students of a health degree
130.	Crossing,	2000	Ineligible study design/ type	A letter to the Editor

131.	Cuite,	2008	Ineligible intervention	Not about prognosis of a health condition
132.	Damhus,	2018	Ineligible study design/ type	Qualitative study design
133.	Edwards,	2002	Ineligible study design/ type	Evidence synthesis not a primary study
134.	Edwards,	2006	Ineligible intervention	Not about prognosis of a health condition
135.	Fagerlin,	2005	Ineligible intervention	Not about prognosis of a health condition
136.	Gaissmaier,	2012	Ineligible outcome	Unusable data
137.	Galesic,	2009	Ineligible intervention	No specific time period in the intervention
138.	Garcia-Retamero,	2009	Ineligible intervention	No specific time period in the intervention
139.	Garcia-Retamero,	2010	Ineligible intervention	Not about prognosis of a health condition
140.	Garcia-Retamero,	2013	Ineligible intervention	Not about prognosis of a health condition
141.	Gong,	2015	Ineligible intervention	Descriptive prognostic information
142.	Grimes,	1999	Ineligible intervention	Not about prognosis of a health condition
143.	Han,	2011	Ineligible intervention	Not about prognosis of a health condition
144.	Haward,	2008	Ineligible outcome	Comprehension was not measured
145.	Henneman,	2020	Ineligible intervention	Not about prognosis of a health condition
146.	Hess,	2011	Ineligible intervention	Not about prognosis of a health condition
147.	Housten,	2020	Ineligible intervention	Not about prognosis of a health condition
148.	Janssen,	2018	Ineligible intervention	Not about prognosis of a health condition
149.	Keller,	2017	Ineligible intervention	Not comparing different visual presentations
150.	Kosslyn,	1989	Ineligible study design/ type	Evidence synthesis not a primary study
151.	Kreuzmair,	2017	Ineligible intervention	Not about prognosis of a health condition
152.	Lobb,	2003	Ineligible intervention	Not about prognosis of a health condition
153.	Mazur,	1990	Ineligible intervention	Not compared to other interventions
154.	Mazur,	1990	Ineligible outcome	Comprehension was not measured
155.	Mazur,	1993	Ineligible intervention	Not compared to other interventions
156.	Mazur,	1996	Ineligible outcome	Comprehension was not measured

157.	Mazur,	1993	Ineligible outcome	Comprehension was not measured
158.	McCaffery,	2012	Ineligible intervention	Not about prognosis of a health condition
159.	Moss,	1989	Ineligible study design/ type	Evidence synthesis not a primary study
160.	Naik,	2012	Ineligible study design/ type	Evidence synthesis not a primary study
161.	O'Donoghue,	2014	Ineligible intervention	No specific time period in the intervention
162.	Oken,	1961	Ineligible intervention	Not about prognosis of a health condition
163.	Perneger,	2011	Ineligible intervention	Not about prognosis of a health condition
164.	Porensky,	2016	Ineligible outcome	Comprehension was not measured
165.	Reading	2020	Ineligible intervention	Not about prognosis of a health condition
166.	Reen,	2018	Ineligible study design/ type	Repeated measures study
167.	Rolison,	2012	Ineligible intervention	Not about prognosis of a health condition
168.	Rolison,	2020	Ineligible intervention	Not compared to other interventions
169.	Schapira,	2006	Ineligible intervention	Not about prognosis of a health condition
170.	Schwartz,	1997	Ineligible intervention	Not comparing different visual presentations
171.	Stone,	2015	Ineligible population	Health professionals or students of a health degree
172.	Sullivan,	2015	Ineligible intervention	Not comparing different visual presentations
173.	Sullivan,	2016	Ineligible intervention	No specific time period in the intervention
174.	Timmermans,	2008	Ineligible intervention	Not compared to other interventions
175.	Waters,	2006	Ineligible intervention	Not about prognosis of a health condition
176.	Waters,	2007	Ineligible intervention	Not about prognosis of a health condition
177.	Waters,	2007	Ineligible intervention	Not about prognosis of a health condition
178.	Wegier,	2019	Ineligible intervention	Not about prognosis of a health condition
179.	Wilson,	1987	Ineligible population	Health professionals or students of a health degree
180.	Yamagishi,	1997	Ineligible intervention	Not about prognosis of a health condition
181.	Zikmund-Fisher,	2011	Ineligible intervention	Not about prognosis of a health condition

182.	Zikmund-Fisher,	2005	Ineligible outcome	Comprehension was not measured
183.	Zikmund-Fisher,	2017	Ineligible intervention	Not about prognosis of a health condition
184.	Zikmund-Fisher,	2011	Ineligible outcome	Comprehension was not measured
185.	Zikmund-Fisher,	2014	Ineligible intervention	Not about prognosis of a health condition
186.	Zikmund-Fisher,	2012	Ineligible intervention	Not about prognosis of a health condition
187.	Whelan, T., et al.	2005	Ineligible study design/ type	Evidence synthesis not a primary study
188.	Shiloh, S., et al.	1989	Ineligible intervention	Health professionals or students of a health degree
189.	Reed, S., et al.	2019	Ineligible intervention	Not compared to other interventions
190.	Perneger, T. V., et al	2011	Ineligible intervention	Not comparing different visual presentations
191.	Nakano, K., et al.	2018	Ineligible intervention	Not compared to other interventions
192.	McNair, A. G., et al.	2010	Ineligible study design/ type	Qualitative study design
193.	Lin, C. Y., et al.	2015	Ineligible intervention	Not about prognosis of a health condition
194.	Levine, M. N., et al.	1992	Ineligible outcome	Comprehension was not measured
195.	Dowen, F., et al	2017	Ineligible intervention	Not about prognosis of a health condition
196.	Baumann, B. C., et al.	2017	Duplicate	
197.	Flynn, D., et al.	2015	Duplicate	
198.	Fraenkel, L., et al.	2016	Duplicate	
199.	Hong, C., et al.	2013	Duplicate	
200.	Kang, S. K., et al.	2018	Duplicate	
201.	Moulton, H., et al.	2018	Duplicate	
202.	Oostendorp, L. J. M., et al.	2017	Duplicate	
203.	Van Maurik, I. S., et al.	2019	Duplicate	
204.	Zikmund-Fisher,	2005	Duplicate	
205.	Edwards	2006	Duplicate	
